# Evolution of Genomic Structures on Mammalian Sex Chromosomes

**DOI:** 10.2174/138920212799860625

**Published:** 2012-04

**Authors:** Yukako Katsura, Mineyo Iwase, Yoko Satta

**Affiliations:** 1 Department of Evolutionary Studies of Biosystems, The Graduate University for Advanced Studies, Hayama, Kanagawa 240-0193, Japan; 2 Center for Promotion of Integrated Sciences, The Graduate University for Advanced Studies, Hayama, Kanagawa 240-0193, Japan

**Keywords:** Ectopic gene conversion, evolutionary strata, inverted repeats, palindrames, recombination suppression.

## Abstract

Throughout mammalian evolution, recombination between the two sex chromosomes was suppressed in a stepwise manner. It is thought that the suppression of recombination led to an accumulation of deleterious mutations and frequent genomic rearrangements on the Y chromosome. In this article, we review three evolutionary aspects related to genomic rearrangements and structures, such as inverted repeats (IRs) and palindromes (PDs), on the mammalian sex chromosomes. First, we describe the stepwise manner in which recombination between the X and Y chromosomes was suppressed in placental mammals and discuss a genomic rearrangement that might have led to the formation of present pseudoautosomal boundaries (PAB). Second, we describe ectopic gene conversion between the X and Y chromosomes, and propose possible molecular causes. Third, we focus on the evolutionary mode and timing of PD formation on the X and Y chromosomes. The sequence of the chimpanzee Y chromosome was recently published by two groups. Both groups suggest that rapid evolution of genomic structure occurred on the Y chromosome. Our re-analysis of the sequences confirmed the species-specific mode of human and chimpanzee Y chromosomal evolution. Finally, we present a general outlook regarding the rapid evolution of mammalian sex chromosomes.

## RECOMBINATION SUPPRESSION AND PAB FORMATION

Suppression of recombination is crucial for sex chromosomal differentiation and for the proper transmission of sex chromosomes to the next generation. In eutheria (placental mammals), suppression of recombination between the sex chromosomes occurs in a stepwise manner. This was first observed by Lahn and Page in 1999 [1], who showed that 19 pairs of X-Y human gametologs (homologs originating from a pair of autosomal ‘proto-sex chromosomes’) can be categorized into four groups according to the extent of synonymous nucleotide divergence between each gametologous pair and the location of corresponding genes on the X chromosome. The average nucleotide divergence in each group differs significantly from other groups and decreases in a stepwise manner according to the group position along the X chromosome. Each group is called a “stratum”. The number of synonymous divergence per site (*d*_s_) of gametologs in each stratum is as follows: *d*_s_ of stratum 1, stratum 2, stratum 3 and stratum 4 are ~ 1.0, ~0.5, ~0.3 and ~0.1, respectively. Based on their *d*_s_ values between gametologs, stratum 3 likely emerged after the divergence between eutherian mammals and marsupials, and stratum 4 likely emerged between prosimian and simian primates [2, 3]. Later, Ross *et al*. [4] found that stratum 5, in which *d* is ~0.08, might have been generated after the divergence of New World monkeys and Catarrhini (Old World monkeys and hominoids). In fact, the nucleotide sequences of the marsupial genome [5] confirmed the absence of strata 3, 4, and 5 on the marsupial X chromosome. The sex chromosomal region containing these strata originated from an autosome, and indeed, homologs of genes in strata 3, 4, and 5 are present on autosomes in the marsupial genome [5,6]. The region corresponding to strata 3, 4 and 5 is added to an ancient sex chromosome (the present marsupial type X chromosome) prior to the eutherian radiation, about 100 million years ago (mya) [7].

**Fig. (1). Nucleotide differences between mammalian AMEL gene regions.**
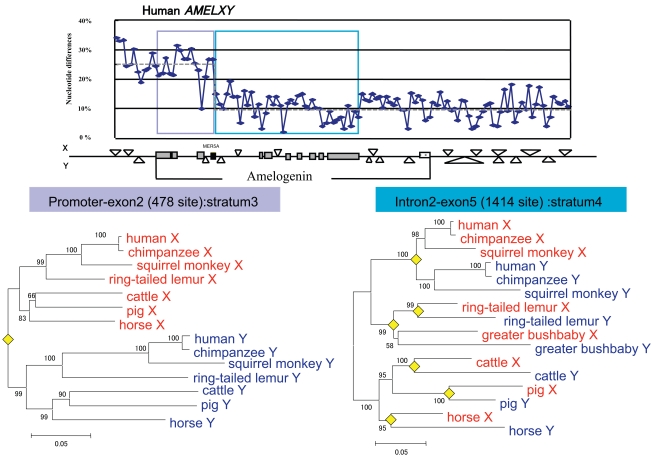
Nucleotide differences between *AMELX* and *AMELY* of humans (top) and the AMEL phylogeny in strata 3 (bottom left) and 4 (bottom right) in mammals are shown. The graph shows the extent of nucleotide difference per site (ordinate) in non-overlapping windows of 100 bp. The abscissa shows the position in *AMEL* genes. A map of *AMELX* and *AMELY* with surrounding regions is shown at the bottom of the graph. Light and dark blue rectangles indicate the regions used for phylogenetic analysis. In the map, the boxes with shading indicate exons of *AMELs*. The filled box shows a transposable element, *MER5*, which is located at the strata boundary. Two phylogenies are shown for strata 3 and 4. The filled diamond in both trees reveals the divergence between the X (red) and the Y (blue) gametologs.

Previous analyses that aimed at identifying strata on sex chromosomes were performed using only the coding sequences (CDS) of genes. However, if each stratum is generated by stepwise recombination suppression, non-coding sequences in each stratum should show the pattern of nucleotide divergence variation similar to that observed for CDS. To clarify this, we compared the available non-coding nucleotide sequences of strata 3, and 4 (including stratum 5) on the short arm of the human X chromosome with that of the corresponding region on the Y chromosome [8]. We confirmed that stepwise differentiation occurred and the presence of clear boundaries among strata 3, 4, and the pseudoautosomal region (PAR) in non-coding regions. One remarkable finding reported in this study is the identification of a boundary between strata 3 and 4 in intron 2 of *AMELX* and *AMELY* (Fig. **1**) in humans. We also confirmed the presence of the same boundary in other mammals [3]. 

This finding is significant in the context of understanding the mechanism by which recombination suppression occurred. One previously proposed, and likely, mechanism is that of an “inversion” on the Y chromosome: the inversion is a possible direct cause to suppress recombination. However, the observation of a boundary in the intron of intact (*i.e.*, functional) *AMELX* and *AMELY* suggests that an inversion cannot explain how the strata 3 and 4 boundary was formed. Instead, an alternative mechanism is likely required. Although we currently do not have a clear idea about this alternative mechanism, some clues might be revealed by investigating the current boundary of recombination suppression, or pseudoautosomal boundary (PAB*)*, which is located between stratum 4 (or 5) and the PAR.

As shown in Fig. (**2**), the present PABs vary among different species, in that the position of the PAB on the X chromosome appears to be order- or species-specific. Currently, the full nucleotide Y chromosome sequences of many species have not yet been published. Therefore, fluorescent *in situ* hybridization (FISH) has been the primary method used to identify the PAB locations and genes nearby the PABs [9]. Interestingly, the *SRY* (Sex determining Region Y) gene is located near the PAB in humans and cats [10]. *SRY *is a gene encoding a transcription factor that is a primary male determining factor in eutherians. The linkage between male determination-related genes, such as *SRY* and RNA binding motif protein (*RBMY*) [3,11], is important in sex determination. Therefore, recombination with X chromosome was likely suppressed by natural selection [12]. When *SRY* or other male determination-related genes are placed into the PAR of the Y chromosome, the position in the PAR would become a PAB. In this regard, it will be interesting to learn more about the function of genes near PABs on the Y chromosomes of different mammals. 

For humans and chimpanzees, the location and nucleotide sequences of PABs on the X and Y chromosomes are available, making it possible to search for clues about how recombination was suppressed. In both humans and chimpanzees, PAB is located in the *XG* gene in both the X and Y chromosomes, and *SRY* is inserted into intron 3 of the *XG* gametolog on the Y chromosome, in an orientation opposite to that of *XG* [13]. Due to this insertion, the human *XG* gametolog (*XGPY2*) on the Y chromosome likely lost the downstream region, from intron 3 to exon 10, and became truncated. Interestingly, the results of previous FISH analyses indicated that the *XG* on the Y chromosome has been duplicated near the centromere (*XGPY*) [13]. This duplication has been confirmed by BLAST analysis of the human Y chromosome sequences. In this article, we will refer to the sequence in the original PAR, including *XGPY2*, as PARY1, and the duplicated sequence as PARY1L. In the human Y chromosome, PARY1L is about 40 kb long. *XGPY* in PARY1L ranges from exon 2 to exon 6 and exhibits 92% similarity to *XG*. On the other hand, *XGPY2* in PARY1 bears an identical sequence from exon 1 to exon 3 to *XG*. The presence of intron 3 to exon 6 in *XGPY* suggests that the duplication might have occurred prior to the loss of exons 3 to 10 in *XGPY2*. The similar duplication of an approximately 40-kb region was also observed in the chimpanzee Y chromosome [14, 15]. The nucleotide divergence between the PARY1 and PARY1L sequences in humans and chimpanzees is 0.087 ± 0.002 and 0.078 ± 0.001, respectively, suggesting that the duplication occurred concurrently with the formation of stratum 5 (~0.08 of the nucleotide divergence between X and Y gametologs) that is located adjacent to PARY. The nucleotide divergence between the X and Y chromosomal regions also indicates that the duplication occurred before the divergence of Old World monkeys and hominoids. If this duplication triggered the formation of the present PAR, then it would be predicted that the PAB in macaques is also located in *XG,* whereas the PAB might be located in different regions in New World monkeys. A detailed examination of PARY1 and their flanking regions in Old World and New World monkeys will be necessary to fully understand the relationship between the duplication and suppression. 

## GENE CONVERSION BETWEEN THE X AND Y CHROMOSOMES

**Fig. (4). Nucleotide differences in the human KAL gene region and corresponding regions in primates.**
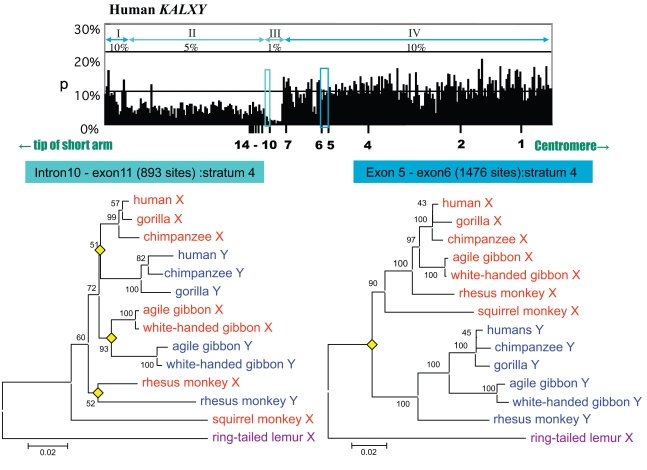
Nucleotide differences per site in a low *p-distance* region (top) in the human *KAL* gene and its flanking region, as well as the phylogeny of regions III and IV of primates (bottom). Bars at the bottom of the top graph represent exons in *KALX*. The tree was constructed using Neighbor joining based on the *p-distances* of *KAL* genes from several primates. The ring-tailed lemur *KALX* sequence was used to root each tree. The yellow diamond represents the divergence between *KALX* and *KALY*.

Except for PAR, genetic exchange between X and Y gametologous sequences, including non-coding regions, should only be observed rarely due to the suppression of recombination. However, a ~100-kb region is found to have significantly low nucleotide divergence compared with neighboring regions in the stratum 4 [3, 4,16]. Fig. (**3**) shows the levels of nucleotide divergence between the human sequence along the short arm of the X chromosome and its gametologous sequence on the Y chromosome. Stepwise changes in nucleotide divergence along the chromosomal arm are observed. The region with a divergence of 0.1% corresponds to *PAR*, the 8% divergent region corresponds to stratum 5, the 12% divergent region corresponds to stratum 4, and the 25% divergent region corresponds to stratum 3. Notably, there is a sub-segment with a significantly low extent of nucleotide divergence (1–5%) in the 12% region compared with the divergence at neighboring sequences. This sub-segment contains the *KALX/Y* and *VCX/Y* genes (see enlargement of *KAL*-coding and its flanking region shown in Fig. **4**). This sub-segment can be further divided into four smaller regions (regions I to IV) corresponding to differences in nucleotide divergence. Regions I and IV have approximately 10% nucleotide divergence, whereas regions II and III have 5% and 1% divergence, respectively. Considering that both the telomeric and centromeric parts of regions II and III show 10% divergence, a decrease in the mutation rate is unlikely, rather, frequent gene conversions likely occurred in these regions. To test this idea, phylogenies for regions III and IV were constructed using other primate sequences, including lemurs and New World monkeys (Fig. **4**). As expected, the tree corresponding to region IV shows a divergence of X and Y sequences prior to the divergence of simian primates, whereas the topology of the tree for region III revealed at least three independent gene conversions between X and Y sequences in the lineage leading to Old World monkeys, gibbons, and other hominoids. Further analysis revealed evidence for seven additional but independent X-Y conversion events during the evolution of Catarrhini [16]. The estimated rate of conversion is approximately 0.1/gene /million years (myr), assuming that the divergence of Old World monkeys, gibbons, orangutans, gorillas and chimpanzees from humans occurred 23, 20, 13, 7 and 6 myr, respectively [17,18].

The ongoing gene conversion between the *X* and *Y* can be observed between *KALX* and *KALY* in extant human populations. Since *KALY* is a pseudogene, whereas *KALX* is functional, conversion of *KALY* to *KALX* can potentially destroy the functional *KALX*, a phenomenon that can result in Kallmann syndrome. Kallmann syndrome is a genetic disease characterized by a deficiency of gonadotropin-releasing hormone (GnRH), resulting in a decreased function of the glands producing sex hormones. In one patient with Kallmann syndrome, exon 14 in *KALX* was replaced by the homologous segment in *KALY* [19].

A second example of ongoing gene conversion events in humans are those that occurred between *VCX* and *VCY*, which was inferred from shared SNPs between the two genes [20]. *VCX *and *VCY* are located near the end of the low nucleotide divergence region. Phylogenetic analysis of the genes also reveals that gene conversion has occurred in both chimpanzees and humans, as well as in an ancestral population of both species [16, 21]. The overall rate of X to Y conversion was estimated to be 1.8 ( 10^-7^ /bp/year for *VCX* and *VCY* [20]. Because the size of these genes is approximately 1.5 kb, the conversion rate is likely 3 ( 10^-4^ /gene/year, which is much higher than the rate observed for *KALX*/*Y*. 

The region of frequent gene conversion is confined to the ~100-kb region. Frequent conversion in this region might be due to the presence of sequence motifs or “hot spots” for non-allelic homologous recombination (NAHR) [22]. In fact, NAHR motifs have been conserved in regions flanking human *VCX* and *VCY* [23], as well as in *VCX/Y *of chimpanzee and macaque *VCX*. The presence of NAHR in primate *VCX/Y* might promote more frequent gene conversion than what is observed in *KALX/Y*. On the other hand, the presence of a LINE element in *KALX/Y* sequences might also promote gene conversion between the two genes [16].

## EVOLUTION OF PALINDROMES ON SEX CHROMOSOMES

Palindromes (PDs) are composed of repeated sequences located in opposite directions, *i.e. *inverted repeats (IRs) with an interval of unrelated sequences [24]. Each repeated sequence is called an “arm” and a pair of arms forms a “stem” in a PD. A sequence sandwiched by two IRs forms is referred to as a “loop”. Warburton *et al*. [24] found many IRs on the X and Y chromosomes, which are likely to form PDs. The high level of nucleotide similarity between arms of the PDs appears to be due to gene conversion between them rather than recent divergence [24]. Interestingly, genes in IRs on the X or Y chromosome tend to be members of gene families that are mainly expressed in the testis and cancer cells [24]. However, we note that the overall gene content in PDs on the X and Y chromosomes is quite different. On the X chromosome, each PD is usually composed of members from a single gene family, whereas on the Y chromosome, each PD often contains members from several different gene families.

One example of genes, found on the human X chromosome PD, is the melanoma antigen A (*MAGE-A*) gene subfamily, which is located at around 150 Mb, near the tip of the long arm of the chromosome. The PD is about ~100 kb in size and seven genes and pseudogenes are located on this PD: *MAGE-A2*, *A2B*, *A3,* *A6*, *A12*, *psA* (a *MAGE*-*A* pseudogene) and *psAL* (‘*MAGE-A *pseudogene-like’) [25]. The *MAGE-A* subfamily is specific to eutherian mammals, and phylogenetic analysis shows that the *MAGE-A* members in a given eutherian species form a species-specific cluster. However, PDs that include a *MAGE-A* gene cluster have not been detected in eutherian mammals other than primates, especially other than Catarrhini. Among the seven members of the human *MAGE-A* family, *A12* is located in a loop region, and the *A2* and *A2B*, *A3* and *A6*, and *psA* and *psAL* pairs are located on symmetrical positions on the arm of the PD. Moreover, nucleotide sequences of the *A2* and *A2B *pair*, *and those of the *psA *and *psAL* pair are almost identical, whereas those of *A3* and *A6* are not. The underlying biology that explains the divergence between *A3* and *A6 *has been discussed in another paper [25]. In brief, *MAGE-A* encodes epitopes that work in cancer immunity and negative selection against homogenization appears to operate to maintain a variety of epitopes among cancer cells. Negative selection against homogenization of sequences is also observed in immunoglobulin genes [26].

**Fig. (5). Duplicated units and specific deletions in humans, macaques, and an inferred common ancestor.**
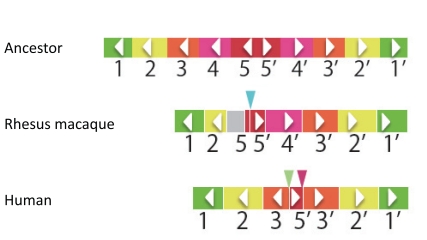
The arrangement of seven duplicated units of palindromes (PDs) in humans and macaques, as well as an inferred arrangement in a common ancestor. Same-color rectangles indicate regions with a high level of similarity in a conspecific genome and orthologs between different species. An arrowhead in each rectangle indicates the direction of the unit. A triangle indicates the position of a deletion. A gray rectangle represents a region in which the nucleotide sequence has not yet been determined. The arrangement present in an ancestor is parsimoniously inferred from comparison of humans and macaques.

Based on the level of nucleotide divergence among members at asymmetrical positions, the divergence among *MAGE-A* members in humans is estimated to have occurred in a stem lineage of Catarrhini. The estimated time of human PD formation on the X chromosome predicted the presence of an orthologous PD in the macaque genome. As expected, a region containing several *MAGE-A* genes was identified in the Rhesus macaque genome. However, orthologous relationship between macaque and human *MAGE-A* genes is not obvious from the extent of nucleotide divergence between homologous regions and phylogenetic analysis: the divergence between a pair of possible orthologs is unexpectedly large [25]. Furthermore, the form of PD in macaques is expected to differ from that in humans: in macaques, high extents of sequence similarity between pairs among *MAGE-A* genes are not observed, except for the *psA* and *psAL* pair, which are located at the outermost part of the PD. Thus, the predicted PD in macaques has a relatively short stem and large loop compared with that in humans. When we compared the PDs of the two species, we found species-specific deletions (Fig. **5**). Interestingly, these species-specific deletions were observed not only in these two species, but also in other primates, such as chimpanzees and orangutans. Although the nucleotide sequences of the corresponding region in chimpanzees and orangutans are not yet complete, the species-specific deletion was inferred from a comparison of duplicated units in each species. The species-specific deletions are indicative of PD instability.

On the human Y chromosome, there are eight palindromes on the long arm of the chromosome. We examined the nucleotide divergence between different gene members in different PDs or at asymmetrical positions in a single PD, and concluded that the majority of gene members in PDs were duplicated in the stem lineage of Catarrhini (~30–50 mya) [21]. Notably, the formation of PD on the Y chromosome coincides with that on the X chromosome. Because the formation of PD is dependent on the presence of IRs, this nearly simultaneous formation of PDs on X and Y chromosomes indicates simultaneous segmental duplication events in the ancestor genome, which might be related to a burst of LINE activity in the primate genome [27].

**Fig. (6). Palindromes on the human and chimpanzee Y chromosomes.**
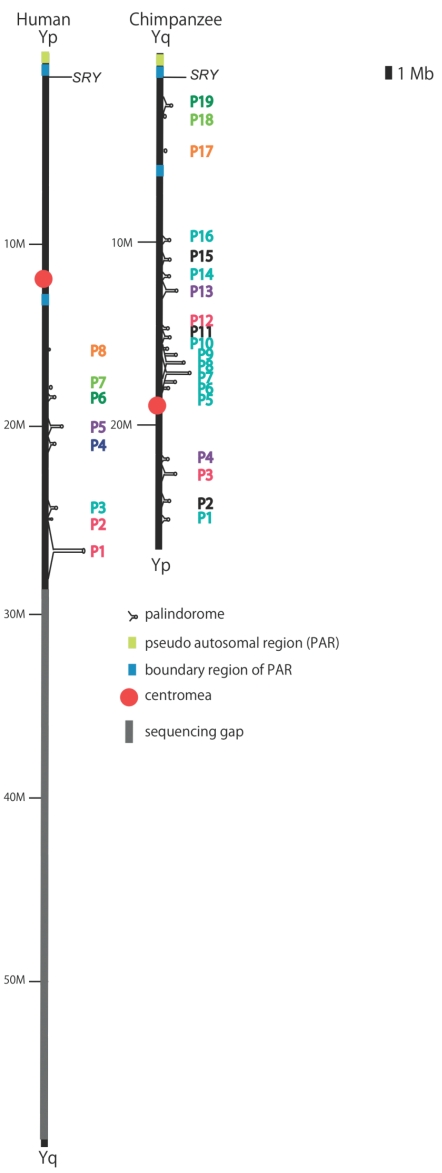
Palindromes (PDs) on the human (left) and chimpanzee (right) Y chromosomes. The blue bars indicate the position of *PARY1* and *PARY1L*. The same-colored number on the right side of each chromosome indicates homologous PDs.

Recently, the nucleotide sequences of the chimpanzee Y chromosome were independently reported by two groups [14, 15]. Both analyses revealed drastic changes in the genomic structures in chimpanzees and/or humans following the divergence of the two species. It is remarkable that a large number of PDs (*i.e.*, 19 PDs) are distributed on the chimpanzee Y chromosome, whereas only eight palindromes have been maintained in humans [2,15]. To investigate the evolutionary mode of structures on the sex chromosome, we examined the origin and relationship between these PDs in chimpanzees and humans. To distinguish human and chimpanzee PDs in the following discussion, we will refer to the PDs in humans as HPD1 to HPD8, and those in chimpanzees as CPD1 to CPD19 (Fig. **6**). The eight human PDs and 19 chimpanzee PDs can be classified into three groups by their origin: human-specific, chimpanzee-specific, and shared PDs between the two species. A simple dot-plot analysis between the two chromosomes revealed that HPD 6, 7, and 8 are orthologs of CPD 19, 18, and 17, respectively [14, 15]. Though HPD7 and CPD18 do not contain any genes, these sequences are unique in the genome and are well conserved between the two species. It will be interesting to determine whether this PD structure is conserved in other primates. If the PD sequence is conserved in orangutans or macaques, for example, then the conservation might indicate that these nucleotide sequences have biological significance in these primates. 

In contrast to the conserved evolutionary mode of HPD 6, 7, 8 and CPD 19, 18, 17, the remaining PDs exhibit rather divergent mode. HPD4 in humans and CPD 2, 11, and 15 in chimpanzees are specific to each species, whereas the remaining PDs share ancestry (Fig. **6**). HPD4 contains a set of 16 members from seven gene families (*OFD1*, *USP9Y*, *XKRY*, *TCEB1*, *HSFY*, *TTY*, *TRAPPCA2*) and among these 16 genes, *HSFY1/HSFY2* exist only in HPD4 and thus they have been lost specifically in chimpanzees [15]. Considering that *HSFY*s are observed widely in mammals, the gene may play an important role in spermatogenesis [28, 29]. It is interesting whether or not there is a gene that can compensate a function of *HSFY*s in chimpanzees. 

On the other hand, CPD 2, 11, 15 are specific to chimpanzees and all contain three pairs of genes: C-terminal-binding protein 2-like (*CtBP2L*), keratin type I cytoskeletal 18-like (*KRT18L*), and carboxy-terminal domain RNA polymerase II polypeptide A small phosphatase 2-like (*CTDSP2L*). The nucleotide sequences of these PDs in chimpanzees show a quite high level of similarity to one another. It is remarkable that nucleotide sequences not only among CPD 2, 11, and 15, but also those between other PDs on the short and long arm of the Y chromosome show a high level of similarity [15]. Although only partial similarity between different PDs is observed in humans [21,30], the similarity between the entire PDs in chimpanzees was a new finding [15], indicating that entire PDs were recently duplicated in chimpanzees.

Hughes *et al*. [15] proposed that CPD 2, 11 and 15 arose from an autosome, because homologs of genes in the PDs are located on autosomes. If this is true, we can calculate the synonymous divergence of these genes on CPDs and autosomes, and estimate the emergence time of these PDs. Three genes on CPDs, *CtBP2L*s, *KRT18L*, and *CTDSP2L*, are nearly identical (~1% nucleotide difference) compared with the corresponding sequences on the chromosome 7. Moreover, all of the homologous genes are located in a region of ~30 kb, near the tip of the short arm of chromosome 7. The extent of nucleotide differences suggests that the formation of these specific CPDs occurred specifically in chimpanzees after the divergence of chimpanzees from humans.

The remaining PDs in humans and chimpanzees share common ancestry. For example, HPD1 and HPD2 show partial similarity to CPD3, HPD5 shows similarity to CPD4 and CPD13, and HPD3 shows similarity to CPD1, CPD5-CPD10, CPD14 and CPD16. HPD3 and nine CPDs contain both *RBMY *and chromodomain protein Y –linked (*CDY*) genes and these genes seem to have been multiplicated specifically in chimpanzees through amplification of CPDs. 

Instability of Y chromosome appears to be evident from the structural differences found between species as closely related as humans and chimpanzees. By comparing the complete Y chromosome sequence of different primates as well as other mammals, this and other interesting questions about the evolutionary mode of Y chromosome may be addressed in the future. 

## FUTURE PERSPECTIVES

We would like to specify three main points regarding evolution and the origin of genomic structures on the sex chromosomes. First, we focus on the bias in the number of IRs and PDs on the X and Y chromosomes. Here, we presented a comparison of the numbers and positions of IRs and PDs between the human and chimpanzee genomes. It remains to be determined whether the bias observed in these two species has been evolutionarily conserved. In a preliminary study, IRs or PDs have been found on the X chromosome of other eutherian mammals, but interestingly such genomic structures are rare in marsupial and platypus X chromosome. IRs and PDs have not been detected even on platypus chromosome 6, a proto-sex chromosome in Therians (marsupials and eutherians). If they are truly absent, the presence of PDs on sex chromosomes might be specific to the eutherian lineage. In fact, mouse X chromosome has its own IRs and PDs that are not shared with humans. Furthermore, the genomic structures in the eutherian X chromosome predominantly contain genes in the cancer testis antigen (*CTA*) gene super-family, exemplified by *MAGE* gene family members. The origin of *CTA* family genes is not fully understood. The relationship between genomic structures like PDs and the origin of *CTA* family genes is an interesting topic to be further explored.

The second point to be determined is the biological importance of IRs or PDs on sex chromosomes. On the Y chromosome, it is thought that IRs or PDs might have developed to compensate for the lack of recombination by providing a mechanism for intra-chromosomal gene exchange. However, this reason does not hold true for the presence of IRs or PDs on the X chromosome. The X chromosome experiences recombination and therefore, compensating for the lack of recombination by gene conversion between PD sequences cannot explain the presence of PDs on X chromosomes. If it is so, the biological significance of having PDs on the X chromosome should be investigated from other viewpoints. 

The third point we wish to address is the mechanism of recombination suppression between sex chromosomes. The presence of strata, or stepwise suppression of recombination along the sex chromosomes, is also observed in chickens, dioecious plants and smut fungi [31-33]. In chickens, the sex chromosomes are designated as Z and W, with the ZW genotype corresponding to females and ZZ to males. The chicken sex chromosomes are characterized by nonlinear strata, *i.e.*, the extent of Z-W divergence does not correspond to the location of genes on the Z chromosome [31]. In a dioecious plant, *Silene latifolia*, the extent of synonymous divergence has increased in proportion to the distance from *PAR* on the sex chromosome. Although the increase is linear, a distinct stratum has not been observed, suggesting that steps suppressing recombination between the sex chromosomes in this plant are still progressing [32]. In the smut fungus *Microbotryum violaceum,* the synonymous divergence between sex chromosomes is ~10%, suggesting that sex chromosomes recently differentiated in this species. However, a gradual increase of nucleotide divergence towards a sex-determining (mating type-determining) gene is clearly observed [33]. 

Suppression of recombination appears to be a crucial step for sex chromosome differentiation in many organisms. It will be interesting to determine whether or not there is a general mechanism of recombination suppression. We know that PAB in the X chromosome is specific for each species. Thus, specific rearrangements at PAB on the Y chromosome should be examined*, *since they might provide new insights about a common molecular mechanism, other than inversion, that could lead to suppression of recombination between sex chromosomes.

Addressing these questions will shed light on the mechanism of sex chromosomal evolution and will hopefully reveal the broader evolutionary significance of genomic structures like IRs or PDs. 

## Figures and Tables

**Fig. (2). Comparative gene map of pseudoautosomal boundaries among selected mammals.**
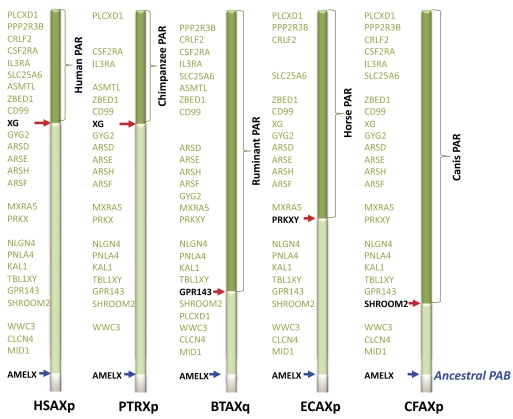
Gene maps of the human (HSA), chimpanzee (PTR), cow (BTA), horse (ECA) and dog (CFA) pseudoautosomal boundaries (PABs) and adjacent regions are shown. The positions of PABs and *AMELX* (ancient PABs) are indicated by red and blue arrows, respectively. The gene order and contents of the stratum (shown in light green) adjacent to PAB and PAR (dark green) have been well conserved in mammals.

**Fig. (3). Summary map of nucleotide differences between the human X and Y chromosomes.**
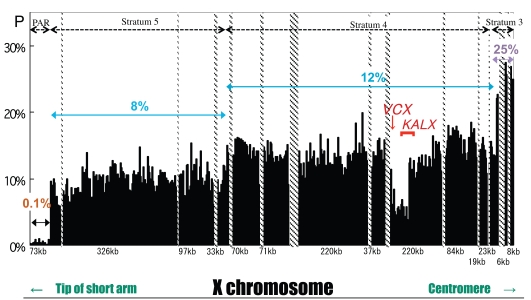
Nucleotide differences per site (ordinate) between the short arm of the human X chromosome and the corresponding region in the Y chromosome. The region is divided into four sub-regions according to the number of nucleotide differences (*p-distance*). From the tip of the short arm (abscissa), the four regions have *p-distances* of 0.1%, 8%, 12%, and 25%, respectively. The *p-distances* were calculated for nonoverlapping windows of 1 kb. In the middle of the region with a *p-distance* of 12%, there is a smaller region with significantly lower pdistance than in neighboring regions. A red arrow and a red square bracket show the position of *VCX* and *KALX* genes, respectively.
